# An Improved Method for Temporary Suture Medialisation of the Middle Turbinates following Endoscopic Sinus Surgery

**DOI:** 10.1155/2018/9093545

**Published:** 2018-09-02

**Authors:** Eugene Wong, Narinder Singh

**Affiliations:** Department of Otolaryngology, Head & Neck Surgery, University of Sydney/Westmead Hospital, Sydney, NSW, Australia

## Abstract

**Background:**

Middle turbinate (MT) lateralisation with adhesion formation (MiTLAF) is a common complication following endoscopic sinus surgery, frequently resulting in surgical failure, persistence of preoperative symptoms, and delayed secondary complications. Packing materials, splints, or spacers reduce the risk of MiTLAF but often result in postoperative nasal obstruction and discomfort, along with reduced access for irrigation. Temporary suture medialisation of the MTs reduces the risk of MiTLAF and prevents the problems encountered with packing, splints, or spacers. However, the techniques described in the literature are technically challenging and often ineffectual.

**Methods:**

We describe a method of suture placement that provides a secure temporary MT medialisation, without the technical challenges of traditional techniques, using a 4-0 Monocryl (Poliglecaprone 25, Ethicon, Somerville, NJ, USA) suture on a 19 mm precision point reverse cutting PS-2 curved needle. We review 25 consecutive patients undergoing sinonasal procedures with our new technique and assess for MiTLAF.

**Results:**

In our cohort, only one patient experienced MiTLAF which was not clinically significant.

**Conclusions:**

Our method is simple, easy to perform, and highly effective and prevents adhesion formation without the need for postoperative splints or packing.

## 1. Background

Middle turbinate lateralisation with adhesion formation (MiTLAF) is one of the commonest complications following endoscopic sinus surgery, occurring in up to 35% of cases [1], and frequently resulting in surgical failure with persistence of preoperative symptoms [2]. Delayed secondary complications related to MiTLAF include frontal recess obstruction, mucocoele formation, and higher rates of revision surgery [2, 3]. Factors likely to increase the risk of MiTLAF include the presence of matched mucosal abrasions on the lateral surface of the middle turbinate (MT) and the lateral nasal wall, mucosal abrasions at the axillary region of the MT, resulting in lateralising contractile forces during wound healing, MT destabilisation secondary to posterior ethmoidectomy or sphenoid access procedures, and postoperative blood clot formation [4, 5].

The risk of MiTLAF may be reduced by temporarily medialising the MT during the early postoperative healing phase. Numerous techniques have been described for temporary medialisation but are compromised by various limitations [2, 4–7].

The placement of packing materials, splints, or spacers between the MT and lateral nasal wall results in postoperative nasal obstruction and discomfort whilst the pack is in situ [8]. Packing materials may result in mucosal injury and delayed return of normal mucociliary function [9]. Nonabsorbable packs must be removed postoperatively, further traumatizing mucosa and causing patient discomfort [9], as well as limiting the use of postoperative irrigation and topical medications. Packs made from absorbable materials prevent the pain of removal associated with nonabsorbable packs. However, whilst present, they similarly contribute to patient discomfort and limit the use of postoperative irrigation and topical medications. In some instances, certain absorbable materials have been shown to increase the risk of adhesion formation [3, 10].

“Bolgerisation” (creating matched mucosal abrasions between the MT and septum) results in permanent adhesions between the MT and septum, potentially compromising olfaction and permanently disrupting nasal airflow dynamics [7].

Absorbable sutures between the MT and septum provide a suitable temporary medialisation of the MT that reduces the risk of MiTLAF, does not impair healing, and prevents the postoperative nasal obstruction and discomfort associated with packs or splints [11, 12]. However, placing and tying the suture within the nose can be technically challenging [2], particularly as most authors advocate tying the knot deep within the nose, close to the MTs [4–6]. This necessitates an “instrument tie” in an area with limited access for instruments. A poorly tied or loose knot will not effectively medialise the MT, thus compromising the function of the suture.

Furthermore, the choice of suture material may influence the outcome. Traditionally, catgut, either plain or chromic, has been used for septoplasty closure. For MT medialisation, however, catgut [2] has weaker tensile strength and dissolves prior to the desired period of medialisation, being completely resorbed at 7-10 days. Similarly, Vicryl Rapide [5] (Ionized polyglactin 910, Ethicon, Somerville, NJ, USA) is useful for septoplasty closure but dissolves too rapidly for MT medialisation, with 50% loss of strength at 5 days and 0% residual* in vivo *strength at 10-14 days [6]. Plain Vicryl (Polyglactin 910, Ethicon, Somerville, NJ, USA) [4], being a polyfilament, does not pass through tissue as easily as a monofilament, resulting in uneven tensioning of a multiple-pass suture. Vicryl has a tendency to disintegrate in the nose postoperatively, rather than dissolve, with sections of the material becoming apparent to the patient.

We describe our method of suture placement that provides a secure temporary middle turbinate medialisation without the technical challenges of traditional techniques, using a suture material that provides high tensile strength and appropriate time to dissolution.

## 2. Methods

### 2.1. Technique

The temporary turbinate medialisation suture (TTM) is placed at the conclusion of a FESS/septoplasty procedure. Septoplasty with removal of a portion of the perpendicular plate of the ethmoid aids in easy passage of the suture through the septum. Without partial bone removal, more anterior cartilaginous placement of the transseptal suture is sometimes necessary. A headlight, needle holder, long Killian's nasal speculum, Adson's forcep, and mosquito forcep are required. A 4-0 Monocryl (Poliglecaprone 25, Ethicon, Somerville, NJ, USA) suture on a 19 mm precision point reverse cutting PS-2 curved needle is used. 

**Figure 1:**
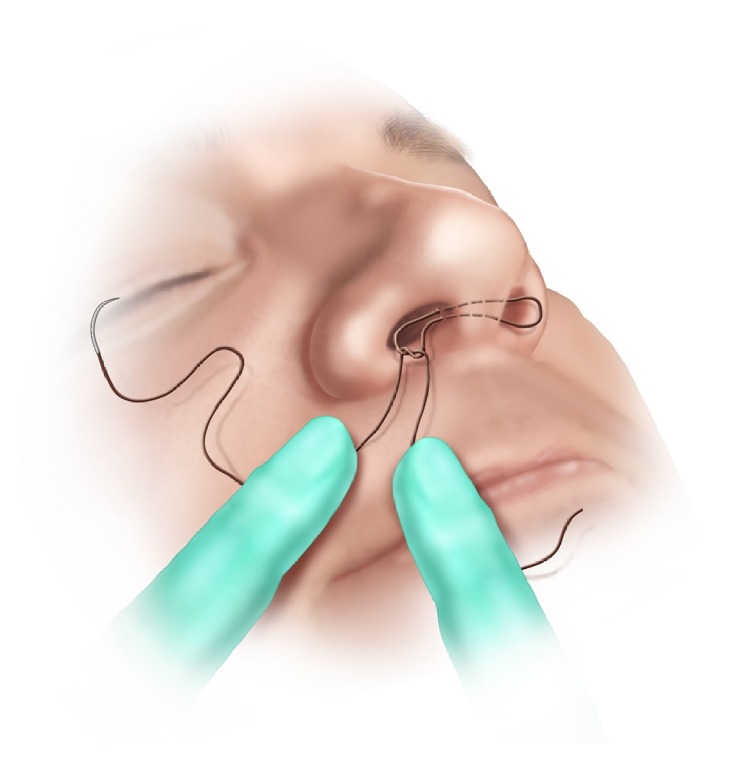
Step 1. A 4-0 Monocryl on a 19 mm precision point reverse cutting PS-2 curved needle is hand-tied at the caudal end of the septum.

*Step 1. Initial Knot*. The initial bite is passed through the septum near its caudal margin, returned back through the septum, and hand-tied to itself, leaving 10-15cm at the nonneedle end, which is clipped with mosquito forceps and passed to the side contralateral to the knot (Figure 1). 

**Figure 2:**
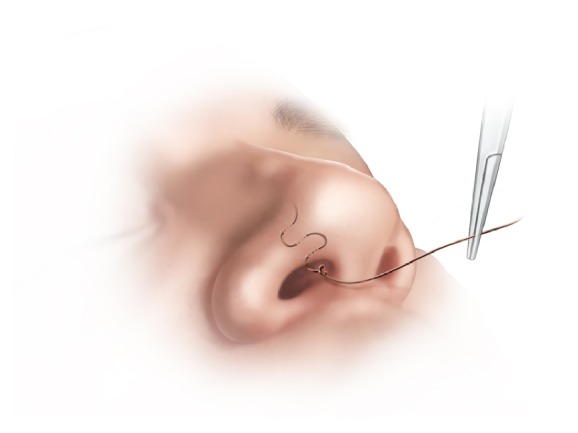
Step 2. A series of passes are made through the septum, each around 1cm posterior to the previous one, until the anterior margin of the MT is reached.

*Step 2. Posterior Running Stitch*. A series of passes are then made through the septum, each pass around 1cm posterior to the previous one, using a long Killian's nasal speculum for visualization, until the anterior margin of the MT is reached (Figure 2). 

**Figure 3:**
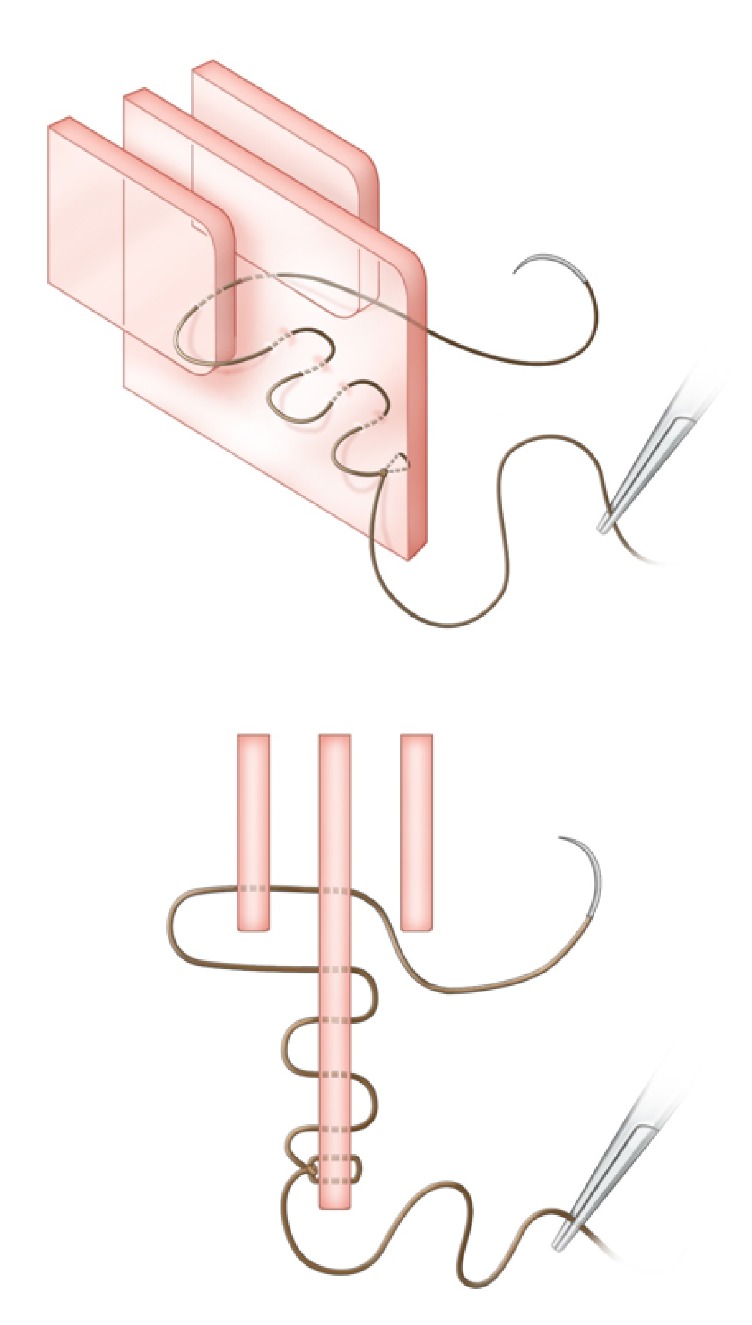
*Oblique and axial views.* Step 3. An initial pass is made incorporating the right MT and the septum alone. The needle tip is then grasped on the left side between the septum and left MT.

*Step 3. Middle Turbinate to Septum*. At this point, if possible, a single pass is made incorporating all three of the MT, the septum, and the contralateral MT. Typically, a single pass suture is technically difficult to achieve. Accordingly, a simpler alternative technique is more commonly used; an initial pass is made incorporating one MT and the septum alone. The needle tip is then grasped on the contralateral side between the septum and contralateral MT (Figure 3).

**Figure 4:**
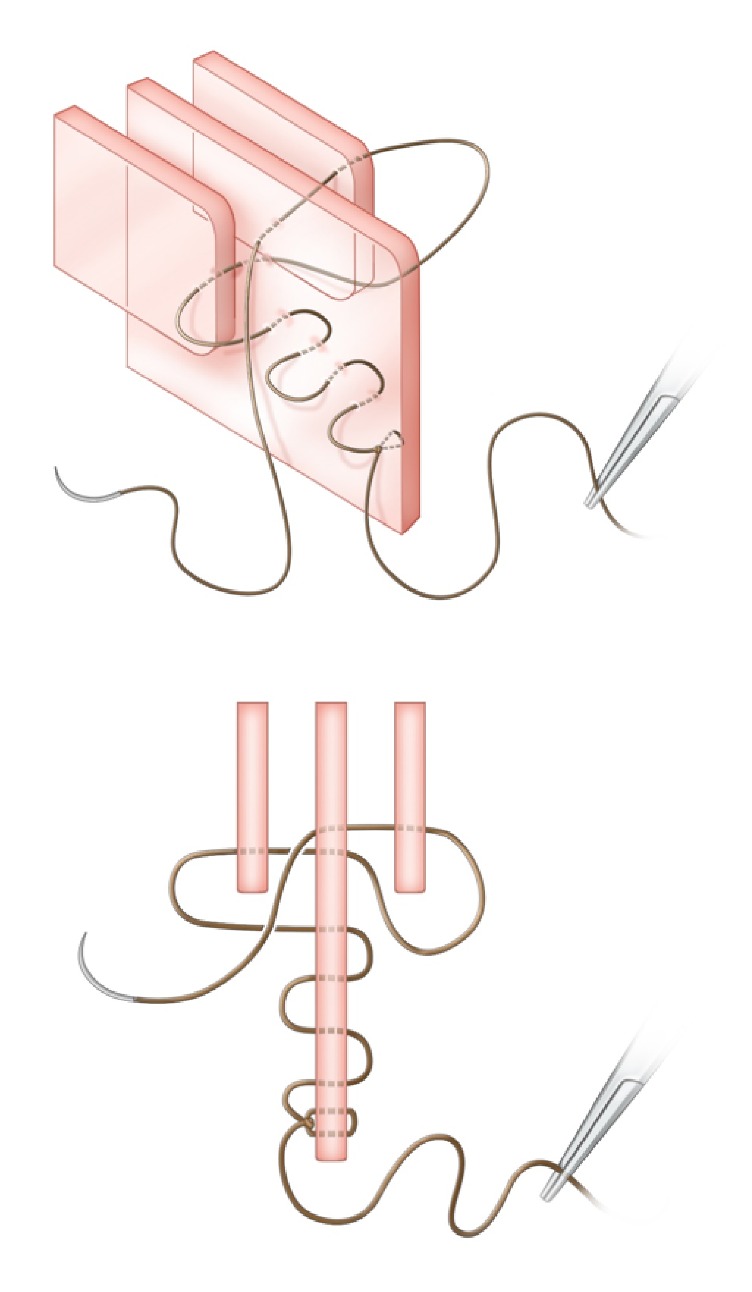
*Oblique and axial views.* Step 4. The needle is reversed and passed through the left MT and septum alone. The needle tip is then grasped on the right side between the septum and right MT and pulled tight (for demonstration purposes, the suture has been drawn loose in this figure and in Figure 5).

*Step 4. Contralateral MT to Septum*. The needle is then reversed and a pass made through the contralateral MT and septum alone. The needle tip is then grasped on the ipsilateral side between the septum and ipsilateral MT and the suture pulled tight, medialising both middle turbinates (Figure 4). If desired, multiple similar passes can be made superiorly or inferiorly, to ensure complete medialisation along the entire height of the middle turbinates. 

**Figure 5:**
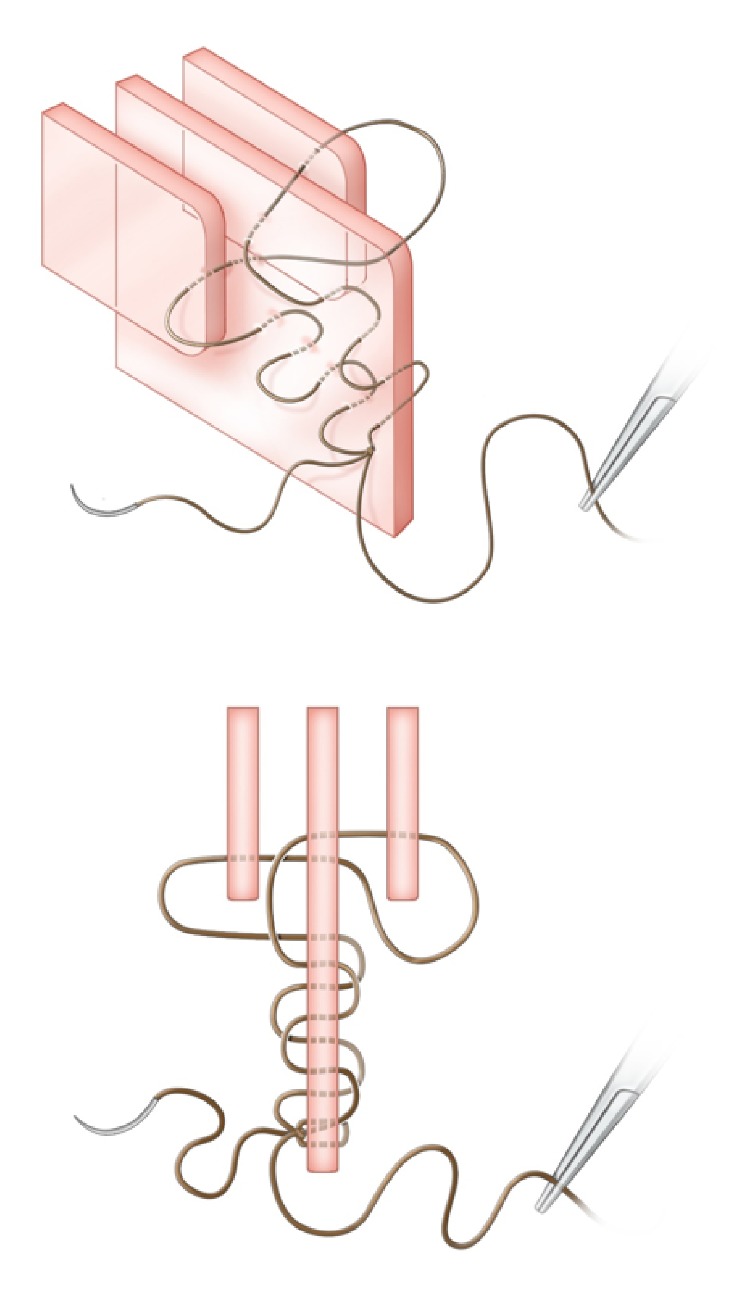
*Oblique and axial views.* Step 5. The suture is then passed anteriorly with 1cm passes.

*Step 5. Anterior Running Stitch*. Whichever of the above methods has been employed, both MTs are now snugly held against the septum. The suture is then passed anteriorly with several 1cm passes until the original knot is reached (Figure 5). 

**Figure 6:**
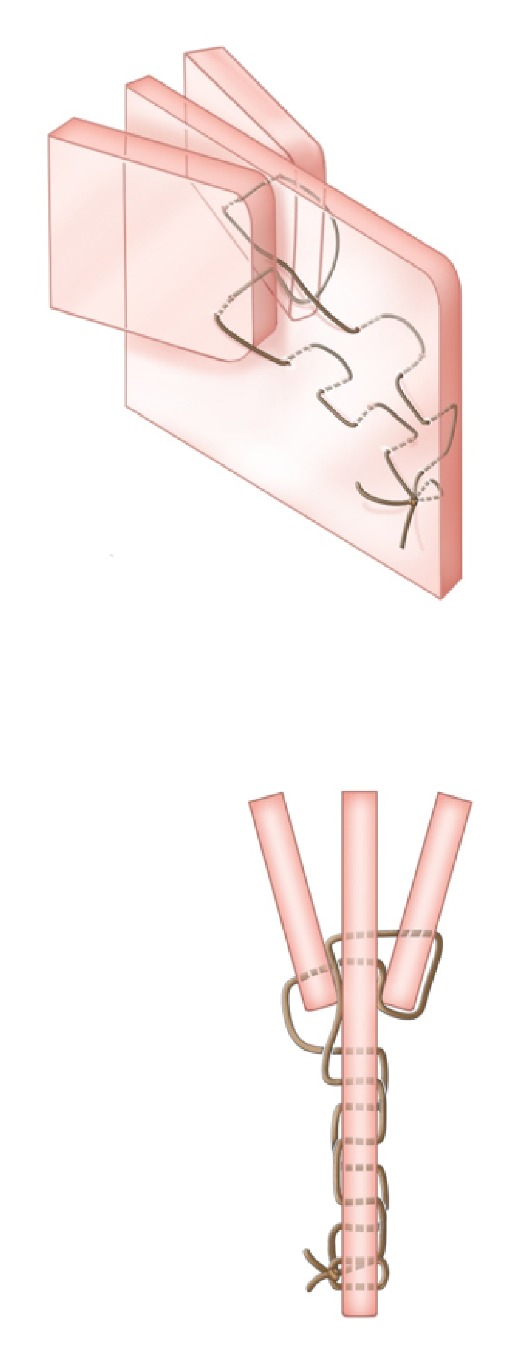
*Oblique and axial views.* Step 6. The suture is hand-tied to the original knot and divided.

*Step 6. Final Knot*. The suture is hand-tied to the original knot and divided (Figure 6).

**Figure 7:**
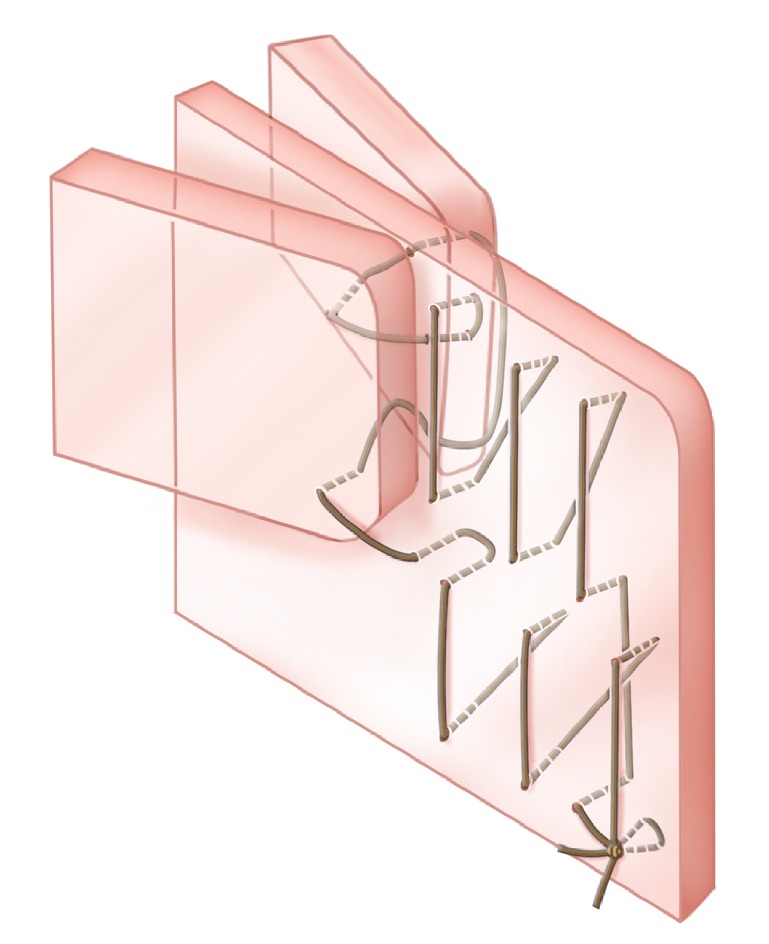
Middle turbinate suturing technique incorporating septoplasty quilting stitch.

The TTM technique may also be incorporated as part of the quilting suture during routine septoplasty closure (Figure 7). Both the Killian or hemitransfixion incisions may be closed using this technique. In our institution, a left-sided hemitransfixion incision was preferred, which was closed using passes through the septum such that the suture lines run posteriorly to anteriorly. The suture can then be tied back to the original knot, or a separate knot, before being divided.

### 2.2. Data Collection

This study was approved by the Western Sydney Local Health District Human Research Ethics Committee.

A retrospective chart review of the author's most recent consecutive bilateral FESS/Septoplasty procedures, incorporating the described suturing technique, was conducted. Primary cases involving chronic rhinosinusitis without polyposis (CRSsP) were included. Other cases, including unilateral procedures, chronic rhinosinusitis with polyposis (CRSwP), tumours, mucocoeles, fungal sinusitis, trauma, access procedures, CSF rhinorrhoea, and revision procedures, were excluded, to reduce confounding factors. Cases were reviewed at 2 weeks and 8 weeks postoperatively using flexible nasendoscopy. Charts were reviewed for the documented presence of MiTLAF at the 2- and 8-week visit.

## 3. Results

Charts for 25 consecutive cases were reviewed between 2012 and 2013. 2 cases (4% of individual sides, 8% of patients) of minor unilateral MiTLAF were identified in the charts, both at 2 weeks postoperatively. In one case, the adhesion was divided in the office at 2 weeks, with no further complications. In the other, asymptomatic case, the adhesion was deemed of limited significance and no intervention was undertaken. The patient remained symptom-free at the last postoperative visit at 8 weeks.

The incidence of MiTLAF identified in this retrospective series may underrepresent the true incidence as the charts may not have recorded every case of adhesion formation and the observer was not independent.

## 4. Discussion

Endonasal suturing is a useful method for temporary middle turbinate medialisation and has been previously described [2, 4–6]. However, the previous literature describes techniques that are technically challenging and awkward to perform, particularly as the knot is tied close to the MT. This may result in a poorly tied or loose knot, which will not effectively medialise the MT, thus compromising the function of the suture. The technique described in this paper is simple and easy to perform, with both knots hand-tied under direct vision at the caudal nasal septum, resulting in a tight suture with effective medialisation.

The technique also overcomes many of the common difficulties encountered regarding suturing within the nasal cavity. In particular, only passing the needle through the MT and then the septum greatly simplifies the procedure when compared to attempting to pass through both MTs at the same time. Instead, the ipsilateral MT is held in place by the septum, making needle passage straightforward. In contrast, attempting to pass from the septum to the contralateral MT often results in the turbinate being pushed away by the needle as there is no countertraction or support.

Furthermore, we describe the use of Monocryl, which has several advantages over materials described in the literature. Monocryl has the benefit of being a monofilament, allowing smooth passage through tissues whilst maintaining good knot-tying and knot-holding properties. It retains 50-70% of its* in vivo* tensile strength at 7 days and 20-40% at 14 days. In clinical practice, it is largely dissolved at 2-4-week follow-up.

Hewitt et al. report a 10.8% adhesion rate following MT suture medialisation. We contend that this relatively high rate may be secondary to their choice of catgut, which lacks tensile strength and dissolves rapidly [6]. We believe that catgut, both plain and chromic, along with Vicryl Rapide, is a suitable material for septoplasty closure and quilting, but not for MT medialisation. MT medialisation requires suture material of high tensile strength, to resist lateralisation forces during healing. Furthermore, the material must last for a sufficient time prior to dissolution to allow healing of mucosa, thus preventing adhesion formation. Monocryl 4-0 most effectively performs these duties. In addition, being a monofilament, it passes easily through tissues with even distribution of tension, compared to polyfilament materials (Plain Vicryl).

## 5. Conclusions

We describe a method of temporary middle turbinate medialisation that is simple, easy to perform, and highly effective and prevents adhesion formation without the need for postoperative splints or packing.

## Data Availability

The data used to support the findings of this study are available from the corresponding author upon request, and the patient's data used to support these findings are included within the article.

## Abbreviations

MiTLAF: Middle turbinate lateralisation with adhesion formation
MT: Middle turbinate
NJ: New Jersey
USA: United States of American
TTM: Temporary turbinate medialisation suture
FESS: Functional endoscopic sinus surgery
CRSsNP: Chronic rhinosinusitis without nasal polyposis
CRSwNP: Chronic rhinosinusitis with nasal polyposis.
